# Long‐term cost‐effectiveness of collaborative dementia care for persons living alone versus those living with a caregiver in Germany

**DOI:** 10.1002/alz.087597

**Published:** 2025-01-09

**Authors:** Bernhard Michalowsky, Moritz Platen, Iris Blotenberg, Stefan Teipel, Jochen René Thyrian, Wolfgang Hoffmann

**Affiliations:** ^1^ German Center for Neurodegenerative Diseases e.V. (DZNE), Site Rostock/Greifswald, Greifswald Germany; ^2^ German Center for Neurodegenerative Diseases (DZNE), Greifswald Germany; ^3^ Deutsches Zentrum für Neurodegenerative Erkrankungen e. V. (DZNE), site Rostock / Greifswald, Greifswald Germany; ^4^ Deutsches Zentrum für Neurodegenerative Erkrankungen e. V. (DZNE) Rostock/Greifswald, Rostock Germany; ^5^ University of Rostock, Rostock Germany; ^6^ Deutsches Zentrum für Neurodegenerative Erkrankungen e.V. (DZNE) and Institute for Community Medicine / University of Greifswald, Greifswald Germany; ^7^ Institute for Community Medicine / University of Greifswald, Greifswald Germany; ^8^ Institut for Community Medicine, University Medicine, University of Greifswald, Greifswald Germany

## Abstract

**Background:**

Previous trials reported that collaborative Dementia Care Management (cDCM) could be cost‐effective in the short term, especially for those living alone. However, long‐term evidence is lacking. Therefore, the study’s objective was to determine the long‐term efficacy and cost‐effectiveness of cDCM in those living alone compared to those living with a caregiver. compared with usual care.

**Method:**

A General Practitioner (GP)‐based, cluster‐randomized‐controlled intervention trial (DelpHi‐MV) was conducted. Participating GP practices were randomly allocated to one of two arms (care as usual or cDCM). Participants were included if they were 70 years or older, living at home, and screened positive for dementia. Participants of the intervention group received a comprehensive needs assessment and individualized interventions by nurses specifically qualified for dementia collaborating with GPs and healthcare stakeholders over six months. Controls received usual care. We conducted a subgroup analyses separating the sample into those living alone versus those living not alone. Health‐Related Quality of Life (SF‐6D), Quality‐adjusted life years, and resource used were assessed at baseline, 12, 24 and 36 months.

**Result:**

428 (n = 303 cDCM, n = 125 usual care) participants were included in the analysis. Based on multivariate regression models adjusted for baseline scores, cDCM was more cost‐effective in PlwD living alone. Compared to controls, the gain in QALY (alone: +0.224 [95% CI 0.03 to 0.42], p = 0.027; not alone: 0.079 [95% CI ‐0.11 to 0.27], p<0.417), savings in costs (alone: ‐3,815 [‐12,573 to 5,982], p = 0.485; not alone: 3,283 [‐3,514 to 11,121], p = 0.307), and the probability of cost‐effectiveness at WTP 40.000€/QALY was significantly higher in PlwD living alone (92% vs 32%). Differences in costs were especially due to a trend for delayed institutionalization in the first and second year for those living alone.

**Conclusion:**

cDCM is cost‐effective in the long term beyond the intervention or short‐term periods, improving patients HRQoL and reducing costs in those living alone. Therefore, should become a health policy priority and translated into routine care practice. Whether cDCM more likely leads to better‐prepared institutionalization should be investigated in future research to support the community‐dwelling living situation as long as possible and make institutionalizations as smooth as possible.

